# A broad-host-range lytic phage vB_VhaS-R18L as a candidate against vibriosis

**DOI:** 10.3389/fmicb.2023.1191157

**Published:** 2023-06-02

**Authors:** Lanlan Cai, Yuan Tian, Ziqiang Li, Yunlan Yang, Chunxiang Ai, Rui Zhang

**Affiliations:** ^1^State Key Laboratory of Marine Environmental Science, College of Ocean and Earth Sciences, Xiamen University, Xiamen, China; ^2^Department of Ocean Science, The Hong Kong University of Science and Technology, Kowloon, Hong Kong SAR, China; ^3^Southern Marine Science and Engineering Guangdong Laboratory, Zhuhai, China

**Keywords:** *Vibrio* phage, phage therapy, genome, biological characteristics, broad host range

## Abstract

Vibriosis is one of the most common bacterial diseases that cause high rates of mortality and considerable economic losses in aquaculture. Phage therapy has been considered as a promising alternative method to antibiotics in the biocontrol of infectious diseases. Genome sequencing and characterization of the phage candidates are prerequisites before field applications to ensure environmental safety. In this study, a lytic phage, named vB_VhaS-R18L (R18L), was isolated from the coastal seawater of Dongshan Island, China. The phage was characterized in terms of morphology, genetic content, infection kinetics, lytic profile, and virion stability. Transmission electronic microscopy indicated that R18L is siphovirus-like, comprising an icosahedral head (diameter 88.6 ± 2.2 nm) and a long noncontractile tail (225 × 11 nm). Genome analysis indicated R18L to be a double-stranded DNA virus with a genome size of 80,965 bp and a G + C content of 44.96%. No genes that encode known toxins or genes implicated in lysogeny control were found in R18L. A one-step growth experiment showed that R18L had a latent period of approximately 40 min and a burst size of 54 phage particles per infected cell. R18L showed lytic activity against a wide range of at least five *Vibrio* species (*V. alginolyticus*, *V. cholerae*, *V. harveyi*, *V. parahemolyticus*, and *V. proteolyticus*). R18L was relatively stable at pH 6–11 and at temperatures ranging from 4°C to 50°C. The broad lytic activity across *Vibrio* species and the stability in the environment make R18L a potential candidate for phage therapy in controlling vibriosis in aquaculture systems.

## Introduction

Vibriosis is a major bacterial disease of aquaculture that is associated with high mortality rates among marine animals and considerable economic losses to the seafood industry (Lafferty et al., 2015). Vibriosis can be caused by a number of *Vibrio* species, among which *V. harveyi* is a notifiable and highly prevalent pathogen in marine environments (Austin and Zhang, 2006; Zhang et al., 2020). Marine vertebrates (mainly fish) and invertebrates (mainly penaeid shrimp) infected by *V. harveyi* show vasculitis, gastroenteritis, eye lesions, and luminous vibriosis. These diseases have severely affected seafood production in Asia and South America, including China, Japan, India, Thailand, Java Island, Philippines, Kuwait, northern Chile, etc. (Austin and Zhang, 2006; Defoirdt et al., 2007). Antibiotics and sanitizers have traditionally been used in the prevention and control of vibriosis in aquaculture (Sano, 1998). However, the overuse of drugs has resulted in antibiotic resistance, chemical residues in aquatic products, a microecological imbalance, and environmental pollution (Defoirdt et al., 2007; Larsson and Flach, 2022). A variety of antibiotic-resistant pathogens have been increasingly reported (Kang et al., 2014; Stalin and Srinivasan, 2016). The emergence of antimicrobial resistance in pathogens highlights the urgent need for alternative therapeutic methods to reduce mortality and minimize the impact on human health and the environment (Defoirdt et al., 2007).

Bacteriophage (or phage) therapy, which has the advantages of specific targeting, self-replication, and low inherent toxicity, has been historically employed as a biological control strategy and has been proposed as an eco-friendly method to control bacterial disease (Defoirdt et al., 2007; Nobrega et al., 2015; Wang et al., 2017; Yen et al., 2017; Gordillo Altamirano and Barr, 2019). To date, a number of studies have reported the use of phage therapy against *V. harveyi* pathogens (Vinod et al., 2006; Shivu et al., 2007; Crothers-Stomps et al., 2010; Wu et al., 2021). Phages infecting *V. harveyi* have been isolated from various environments and tested in terms of their potential application (Vinod et al., 2006; Shivu et al., 2007; Crothers-Stomps et al., 2010; Wu et al., 2021). For example, phage treatment of *V. harveyi-*infected *Penaeus monodon* larvae resulted in higher survival rates (80%) compared with the control larvae (25%) (Vinod et al., 2006). Lytic phages P4A and P4F, isolated from the seawater of an abalone farm, significantly reduced the population of pathogenic *V. harveyi* (Luo et al., 2016). It is now accepted that phage therapy, after careful selection and extensive studies of phage candidates, will eventually become an effective alternative to antibiotics (Defoirdt et al., 2007; Nobrega et al., 2015; Gordillo Altamirano and Barr, 2019; Nachimuthu et al., 2021). However, comprehensive studies must be undertaken in selecting phage candidates because some phages may encode toxins and/or lead to altered bacterial virulence, and others may be inefficient when applied in the field. One such example is bacteriophage VHML (*V. harveyi* myovirus-like), which was shown to confer virulence in various *V. harveyi* strains (Munro et al., 2003). Similarly, two isolated myoviruses were reported to integrate as prophages into the host genome and induce bacteriocin production when infecting *V. harveyi* strains, which excludes their usage in phage therapy (Crothers-Stomps et al., 2010).

In general, the suitability of a particular phage to control bacterial pathogens is determined by the presence of toxic genes, the host range, the length of viral infection, the number of progeny produced, and, importantly, the stability of the phage in the environment (Hyman, 2019). Considering the high degree of phenotypic and genotypic diversity among *Vibrio* pathogens, a phage with a wide host range is potentially valuable. When a disease is caused by a mixed bacterial infection, the use of a broad-host-range phage with the ability to kill multiple strains would be preferable to a mixture of different therapeutic phages (Hyman, 2019). Taking this into consideration, the present study aimed to isolate bacteriophages with a broad host range and evaluate their efficiency as potential biocontrol agents against vibriosis. Different *V. harveyi*-specific phages (a total of 12 phages) were first isolated and screened to determine their host range. On the basis of its broad host range, one of these phages, vB_VhaS-R18L (hereafter, R18L), was selected for further analysis of its genomic and morphological properties, as well as its burst size and latent period. Furthermore, the virion stability of R18L was determined under different temperature and pH conditions to determine its suitability for potential therapeutic applications in the future.

## Materials and methods

### Phage isolation and purification

The host strain *Vibrio harveyi* BYK0632 used in this study was purchased from the National Pathogen Collection Center for Aquatic Animals, Shanghai Ocean University (Shanghai, China). *V. harveyi* BYK0632 was grown in rich organic (RO) medium (1 M peptone, 1 M yeast extract, 1 M sodium acetate, artificial seawater, pH 7.5) at 28°C with shaking at 180 rpm/min. Surface seawater samples for phage isolation were collected in April 2016 at the coast of Dongshan Island (Fujian, China) and filtered through a 0.2 μm membrane. Before being mixed with the host strain, the virus-containing filtrate was concentrated using a 30 kDa cartridge (Millipore, MA, USA) by tangential flow filtration to improve the probability of successful phage infection (Cai et al., 2015, 2019). The concentrated seawater samples were mixed with exponentially-growing *V. harveyi* BYK0632 (OD_600_: 0.1–0.2) using a double-layer agar method according to previous studies (Yang et al., 2017; Cai et al., 2019). After 18–24 h incubation at 28°C, individual lytic plaques were picked from the agarose plate and dissolved in SM buffer (50 mM Tris–HCl, 0.1 M NaCl, 8 mM MgSO_4_, 0.1 g/L gelatin, pH 7.5). This double-layer agar plating was repeated five times to ensure the purity of the phage.

### Preparation of high-titer phage suspensions

To obtain high-titer phage suspensions for morphological observation and genome sequencing, phages were propagated in one liter of *V. harveyi* BYK0632. After cell lysis, the culture was centrifuged at 10,000 × g for 10 min and filtered through 0.2 μm filters to obtain the phage-containing suspension. The phage suspension was precipitated with polyethylene glycol 8,000 (100 g L^˗1^ final concentration) overnight at 4°C and collected by centrifugation at 10,000 × g for 30 min at 4°C. The phage pellet was re-suspended in SM buffer and then concentrated by CsCl (1.5 g/mL in SM buffer) gradient ultracentrifugation (200,000 × g, 4°C, 24 h). The clear phage band was extracted and dialyzed against SM buffer at 4°C.

### Host range

The lytic profiles of purified vibriophages were determined using spot assay (Yang et al., 2017; Cai et al., 2019). Briefly, different phages were challenged against 28 *Vibrio* strains from 12 *Vibrio* species (*V. alginolyticus*, *V. campbellii*, *V. cholera*, *V. harveyi*, *V. inhibens*, *V. mimicus*, *V. owensii*, *V. parahemolyticus*, *V. proteolyticus*, *V. tubiashii*, *V. vulnificus*, and *V. rotiferianus*), which were originally isolated from the aquatic environment and diseased shrimp and fish (Table 1) and purchased from the National Pathogen Collection Center for Aquatic Animals (China). Each of these exponentially growing bacterial cultures was mixed with molten RO agar medium (0.5% w/v agar) and poured onto solid RO agar medium (1.5% w/v agar). After the agarose plates solidified, 5 μL of diluted phage lysate was added onto the surface of each bacterial plate and incubated at 28°C for more than 12 h. The formation of clear plaques where lysates were added indicated successful phage infection of the test strains. Tests were repeated at least three times. One of these isolated phages, R18L, showing a broad host range (see results below), was selected for further characterization.

**Table 1:** Host range of vibriophage R18L (+, infected; −, uninfected).

Species	Strain	Strain type	Infectivity
*V. alginolyticus*	BVA1	Pathogenic	+
*V. alginolyticus*	BVA2	Pathogenic	−
*V. alginolyticus*	20,140,910–1	Pathogenic	−
*V. cholerae*	20,161,020–5	Pathogenic	+
*V. cholerae*	20,160,707–2	Pathogenic	−
*V. harveyi*	20,160,918–11	Pathogenic	−
*V. harveyi*	20,150,916–2	Pathogenic	−
*V. harveyi*	BYK0632	Pathogenic	+
*V. harveyi*	BVH1	Pathogenic	+
*V. inhibens*	3,707	Nonpathogenic	−
*V. mimicus*	20,150,901–2	Pathogenic	−
*V. mimicus*	20,160,921–1	Pathogenic	−
*V. owensii*	3,186	Nonpathogenic	˗
*V. parahemolyticus*	BVP1	Pathogenic	+
*V. parahemolyticus*	BVP2	Pathogenic	−
*V. parahemolyticus*	7D	Pathogenic	−
*V. parahemolyticus*	8D	Pathogenic	−
*V. parahemolyticus*	20,160,623–13	Pathogenic	+
*V. parahemolyticus*	20,160,725–2	Pathogenic	+
*V. parahemolyticus*	4F	Pathogenic	+
*V. parahemolyticus*	20,160,615–5	Pathogenic	−
*V. parahemolyticus*	20,160,707–8	Pathogenic	˗
*V. parahemolyticus*	20,160,719–2	Pathogenic	−
*V. vulnificus*	20,161,213–1	Pathogenic	−
*V. proteolyticu*	3,562	Nonpathogenic	+
*V. campbellii*	3,507	Nonpathogenic	−
*V. tubiashi*	3,833	Nonpathogenic	−
*V. rotiferianus*	3,557	Nonpathogenic	−

### Transmission electron microscopy (TEM)

The morphology of R18L was determined by TEM. In brief, 3 μL of high-titer phage was adsorbed onto a carbon-coated copper microscopy grid for 10 min, followed by negative staining with 2% (w/v) phosphotungstic acid for 1 min. After the grid was air-dried for 30 min, the sample was observed by TEM using a JEM-2100 microscope (JEOL, Tokyo, Japan) at 80 kV. Images were acquired by a CCD image transmission system (Gatan Inc., Pleasanton, CA, United States).

### Lipid test

To determine whether the capsid of R18L contained lipids, a chloroform sensitivity test was conducted (Yang et al., 2017; Cai et al., 2019). Briefly, 1 mL of phage lysate was incubated with 20 μL and 200 μL of chloroform for 30 min at room temperature in the dark. Control aliquots were included without the addition of chloroform. After incubation, chloroform was removed by centrifugation at 5,000 × g for 5 min. The titers of the phage were then determined by a spot assay. Each treatment was tested in triplicate.

### One-step growth curve

The life cycle of R18L was examined by a one-step growth experiment (Middelboe et al., 2010; Yang et al., 2017). Briefly, the freshly prepared phage lysate was added to 1 mL of exponentially growing *V. harveyi* BYK0632 culture with a multiplicity of infection of 0.001 in triplicate, then incubated for 5 min at room temperature (24°C) in the dark for phage adsorption. To remove unabsorbed phage particles, the culture was centrifuged for 5 min (10,000 × g, 4°C) and resuspended in RO medium. This procedure was repeated twice. The suspension was incubated at 28°C in the dark. Samples were taken every 10 min, and the viral abundance was determined by a plaque assay.

### Thermal stability and pH sensitivity

The effects of environmental factors on the phage were determined by testing its thermal stability and pH sensitivity. For the thermal stability test, 1.5 mL of aliquots of freshly prepared phage lysate (~10^7^ plaque-forming units/mL) was incubated at different temperatures (4°C, 24°C, 37°C, 50°C, 55°C, and 60°C) in triplicate. Subsamples were collected at 3, 24, and 48 h, and the phage titer was determined by a plaque assay. For the pH stability test, SM buffer with a pH ranging from 2 to 12 was prepared using HCl or NaOH as required. Then, 100 μL of freshly prepared phage lysate was inoculated and incubated under different pH conditions at room temperature (24°C) in triplicate. Subsamples were collected at 3 h and 24 h, and the phage titer was determined by a plaque assay.

### DNA extraction

Prior to DNA extraction, the high-titer phage concentrate was treated with DNase I and RNase A to remove possible contamination of free host DNA and RNA. The DNase was inactivated at 65°C for 15 min. Phages were lysed with 1.5 μL of proteinase K (100 mg/mL), 10 μL of EDTA (0.5 M, pH 8.0), and 100 μL of sodium dodecyl sulfate (SDS; 10% w/v) at 55°C for 3 h. The phage DNA was extracted with a phenol/chloroform/isoamyl alcohol mixture, which promotes the partitioning of cellular debris into the organic phase, leaving isolated DNA in the aqueous phase. The purified DNA was further precipitated with isoamyl alcohol. The quality of the DNA was checked via agarose gel electrophoresis and analysis using a NanoDrop 2000 Spectrophotometer (Thermo Fisher Scientific, MA, United States).

### Genome sequencing and analysis

The extracted DNA was sheared into 300-bp fragments in a Covaris ultrasonicator (KBiosciences, United Kingdom) before preparing the Illumina paired-end sequencing library using the NEBNext Ultra II DNA library prep kit. The quality and size of the libraries were analyzed using the Agilent 2,100 Bioanalyzer. The concentration of the libraries was determined using the Qubit 2.0 dsDNA HS Assay kit (Life Technologies, Germany). Sequencing was performed on the MiSeq platform (Illumina, San Diego, CA, United States). Raw reads were trimmed and filtered using Trimmomatic v0.36 to remove adaptor sequences and low-quality reads (Bolger et al., 2014). The sequences were assembled using A5-miseq (version 20,150,522). Intergenomic similarities between phages were calculated using VIRIDIC (Moraru et al., 2020). The open reading frames (ORFs) were predicted using GeneMark (Besemer and Borodovsky, 2005) and ORF Finder (Rombel et al., 2002). Gene annotation was performed using BLASTP against the NCBI nonredundant (nr) database^1^ with an e-value <10^˗3^. tRNA sequences in the R18L genome were analyzed by tRNAscan-SE (Chan and Lowe, 2019). The spacer of the R18L genomic sequence was searched against the viral spacer database of IMG/VR (Roux et al., 2021). Phylogenetic analyses of specific genes were performed using the maximum-likelihood method with 1,000 bootstrap replicates and MEGA software (Tamura et al., 2021). The complete genome sequence of vibriophage vB_VhaS-R18L has been deposited in the GenBank database under accession number MT451873.

## Results and discussion

### Biological features of R18L

**Figure 1:**
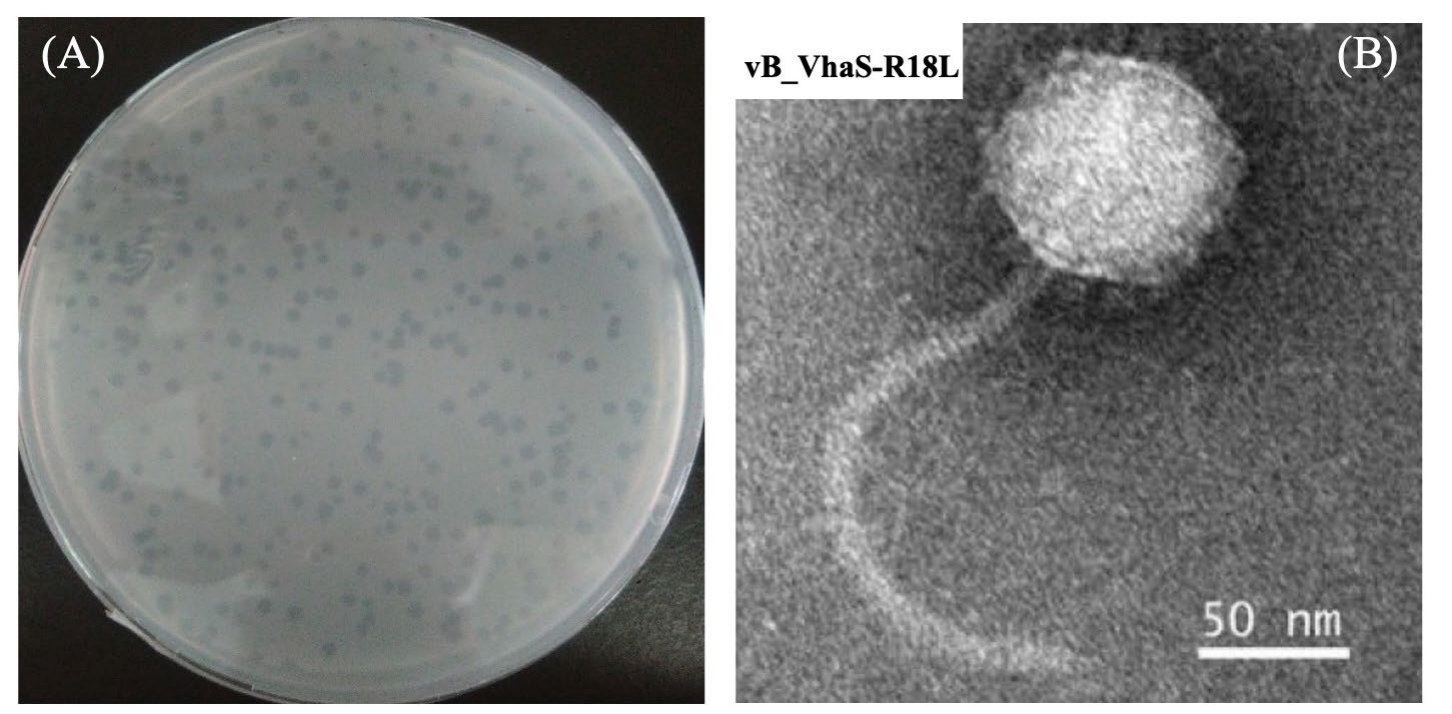
Plaques **(A)** and transmission electron microscopic image **(B)** of vibriophage vB_VhaS-R18L. Scale bar, 50 nm.

In this study, a total of 12 phages against *V. harveyi* were isolated from the coastal surface seawater of Dongshan Island, China. A novel *V. harveyi* phage, designated vB_VhaS-R18L (R18L), showing a broad host range (see below), was selected for further detailed analysis. The lysis of R18L formed semitransparent plaques of 2.0 mm in diameter on host lawn plates (Figure 1A). As shown in the TEM micrograph, R18L has an icosahedral capsid (diameter 88.6 ± 2.2 nm) and a long noncontractile tail (225 ± 2.2 nm in length and 11 ± 1.0 nm in width), being a siphovirus-like phage (Figure 1B). Different vibriophages have been isolated and characterized using *V. harveyi* as the host, with most belonging to siphoviruses (Vinod et al., 2006; Wang et al., 2017; Wu et al., 2021; Droubogiannis and Katharios, 2022; Kang et al., 2022), followed by myoviuses (Surekhamol et al., 2014; Lal et al., 2017) and podoviruses (Thiyagarajan et al., 2011). The reason why siphoviruses are the predominant viral group infecting *Vibrio* is currently unknown. However, as more vibriophage genomes and features become available, we will be better able to assess the abundance and role of different vibriophage groups. The capsid size and tail length of R18L were relatively large compared with those previously reported for siphoviruses with diameters of 40–92 nm and tail lengths of 60–277 nm (Vinod et al., 2006; Crothers-Stomps et al., 2010; Thiyagarajan et al., 2011; Raghu Patil et al., 2014; Stalin and Srinivasan, 2017). The chloroform sensitivity test demonstrated that the infectivity of R18L was not affected by different concentrations of chloroform, suggesting the absence of lipids outside of the R18L capsid. To date, lipids have been considered to be a rare feature for bacteriophages, representing less than 5% of the published isolates (Atanasova et al., 2015; Mantynen et al., 2019).

**Figure 2:**
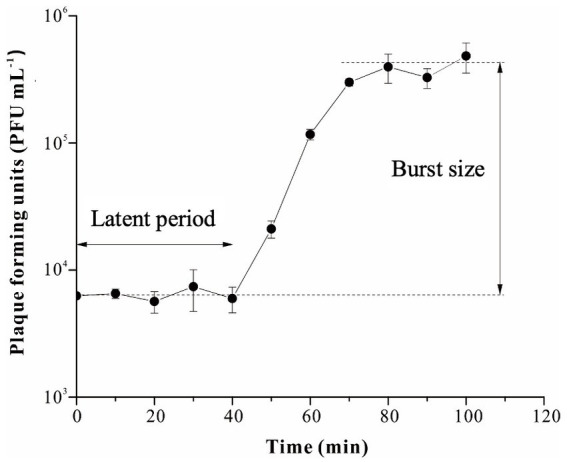
One-step growth curve of vibriophage vB_VhaS-R18L. Data points represent the mean values and standard deviations of three independent experiments.

The latent period and burst size of R18L, as important characteristics of the phage infection process, were determined by the one-step growth curve (Figure 2). R18L exhibited a latent period of 40 min and a rise period of 30 min. The burst size of R18L was calculated at approximately 54 phage particles per infected cell. Recent reports have shown that the latent period and burst size of other *Vibrio* phages were 10–70 min and 2–180 phage particles/cell (Baudoux et al., 2012; Lal et al., 2017; Stalin and Srinivasan, 2017). The latent period and burst size of R18L were within the documented range for vibriophages.

The host range of R18L was tested on 28 *Vibrio* strains, including 22 pathogenic strains isolated from diseased animals and six nonpathogenic strains from seawater (Table 1). Nine (32%) of the 28 *Vibrio* strains tested were lysed by R18L, including strains from *V. alginolyticus*, *V. cholerae*, *V. harveyi*, *V. parahemolyticus*, and *V. proteolyticus*. Of these nine strains, eight were pathogenic. R18L could infect bacteria across at least five *Vibrio* species, even including *V. proteolyticus* and *V. cholerae*, which are not members of the Harveyi clade (Sawabe et al., 2007). Interestingly, two species that are closely related to *V. harveyi* (*V. campbellii* and *V. rotiferianus*), belonging to the Harveyi clade (Sawabe et al., 2007), were not susceptible to phage R18L. Therefore, the genetic similarity between *Vibrio* species does not necessarily correlate with the lytic spectrum of R18L. Previously reported vibriophages have shown different lytic abilities against *Vibrio* species. Phage PW2 infected different strains of *V. harveyi* but not 13 other *Vibrio* species (*V. alginolyticus*, *V. cholera*, *V. campbelli*, *V. logei*, etc.) (Phumkhachorn and Rattanachaikunsopon, 2010). Furthermore, phage SIO-2 could only infect two strains from relatively closely-related *Vibrio* species (*V. harveyi* and *V. campbellii*) when tested against 17 *Vibrio* species (Baudoux et al., 2012). Whereas phages with a broad lytic spectrum have also been reported (Shivu et al., 2007; Thiyagarajan et al., 2011). For example, phages φVh1, φVh2, and φVh3 showed a relatively broad lytic spectrum involving four *Vibrio* species (*V. harveyi*, *V. parahemolyticus*, *V. alginolyticus*, and *V. logei*) (Thiyagarajan et al., 2011). In phage therapy, when bacterial diseases are caused by polymicrobial infections, a therapeutic phage mixture or phages with a broader host range would be needed for treatment (de Jonge et al., 2019; Hyman, 2019). Given that phage cocktails (mixtures) require the individual phage targeting different pathogens to be isolated and studied, broader-host-range phages might be preferable for complex vibriosis. Hence, R18L, possessing a broad spectrum of infectivity against different pathogenic *Vibrio* spp., provides a promising potential biocontrol agent for bacterial diseases in aquaculture. However, the estimation of the efficiency of R18L in treating different pathogens is needed before field applications in the future.

### Genome features of R18L

**Figure 3:**
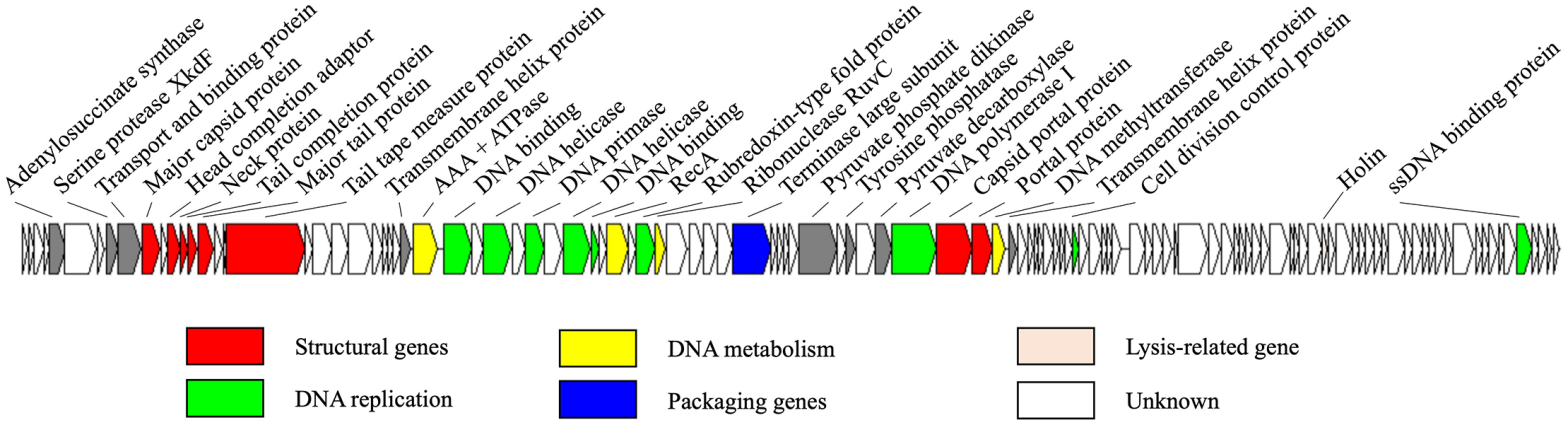
Genome map of vB_VhsS-R18L. Colored arrows represent different putative functions predicted from BLASTP similarity. The direction of each arrow represents the direction of transcription.

The genome of R18L was a double-stranded DNA comprising 80,965 bp and a G + C content of 44.96%. The R18L genome consisted of 118 putative ORFs, of which 31 ORFs (26.2%) have known functions, while the other 87 ORFs (73.7%) were assigned as genes with unknown functions (Figure 3). All of the predicted ORFs are oriented on the positive strand (in a rightward direction). The total gene length of all coding sequences was 76,620 bp, comprising ~94.6% of the genome. Gene annotation using BLASTP (*e*-value <10^˗3^) identified different functional clusters, including structural genes and genes involved in DNA metabolism, DNA replication, DNA packaging, cell lysis, and additional functions (see below). Among the 31 ORFs of known function, eight were related to the structure of R18L, while 15 were associated with DNA replication, metabolism, and packaging, cell lysis (ORF 93). In addition, seven uncategorized ORFs had a wide range of functions, including a serine protease XkdF (ORF 8), which is a protease frequently found in phage genomes. The function of virally encoded serine proteases is currently unknown, but they have been found to be strongly expressed during the late stage of viral infection, suggesting their potential role in virion assembly or maturation (Baum et al., 2021). Tyrosine phosphatase (ORF 53) was involved in signaling by controlling the phosphorylation state of proteins (Walchli et al., 2004). Pyruvate phosphate dikinase (ORF 51) and pyruvate decarboxylase (ORF 55) were two potential auxiliary enzymes that were involved in the host glycolytic metabolism (DelVecchio et al., 2002). No tRNA gene was detected in the R18L genome sequence, suggesting that R18L depended on the translation machinery of its host. Furthermore, R18L did not possess lysogeny-related genes (transposase or integrase, excisionase, and repressor), and no antibiotic resistance genes or virulence factor-related genes were detected in the genome of R18L using the ARDB (Liu and Pop, 2009) and VFDB (Chen et al., 2005) databases, which is beneficial for its potential application in phage therapy.

A nucleotide-based genome comparison revealed that the genome of R18L shared the most similarity to that of *Vibrio* phage SIO-2 (Baudoux et al., 2012), with an identity of 97.33% and a coverage of 99%, consistent with the intergenomic similarity of 96.95% between them based on VIRIDIC calculation (Moraru et al., 2020). However, R18L shows a broader host range than SIO-2. R18L could infect strains of *V. harveyi*, *V. alginolyticus*, *V. cholerae*, *V. parahemolyticus*, and *V. proteolyticus* among 12 *Vibrio* species, while SIO-2 only lysed strains of *V. harveyi* and *V. campbellii* among 17 *Vibrio* species tested. The host range of phages might correlate with variations in their tail-related genes (de Jonge et al., 2019). Three ORFs (ORFs 14, 15, 18) of R18L were identified as tail-related genes, while ORF 15 (major tail protein) of R18L exhibited relatively low identity (93%) with the corresponding gene of SIO-2, which might explain the different host ranges of the two phages. Additionally, seven predicted proteins with unknown function (ORFs 48, 50, 78, 79, 95, 117, 118) of R18L were distinct from those of SIO-2, with 53–88% identity at the amino acid level, which may contribute to the difference in the lytic spectrum of these two phages.

**Figure 4:**
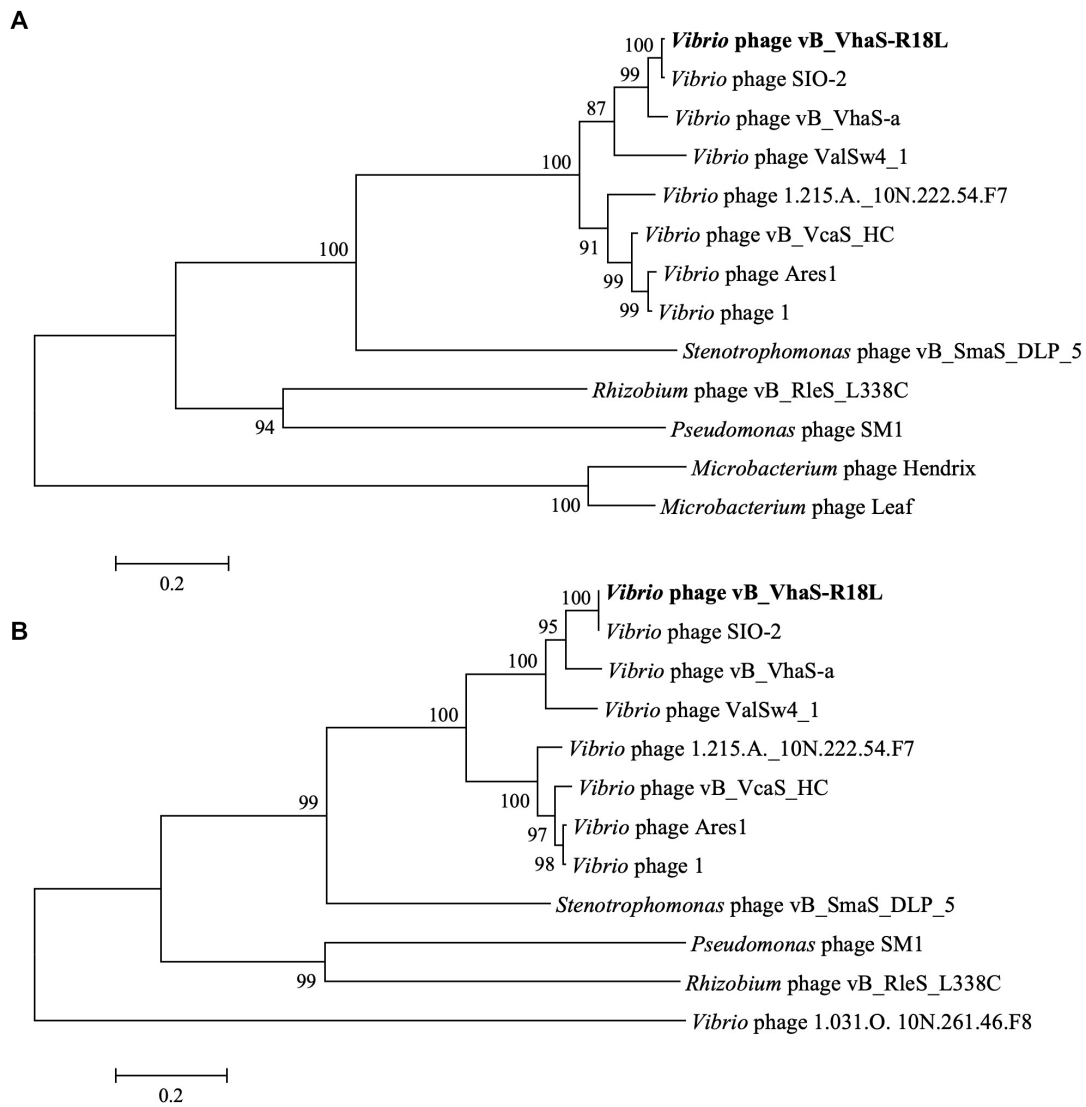
Phylogenetic tree of *Vibrio* phage R18L-related phages based on the amino acid sequences of DNA polymerase I **(A)** and the terminase large subunit **(B)** using a maximum likelihood method with 1,000 bootstraps (percentage value given on nodes). The scale bar represents 0.2 fixed mutations per amino acid position. R18L isolated in this study is shown in bold.

Phylogenetic trees of DNA polymerase I and terminase large subunit (TerL) were constructed to analyze the evolutionary relationships of R18L. As demonstrated in Figure 4, R18L clustered with three vibriophages, SIO-2 (NC_016567.1), vB_VhaS-a (KX198615.1), and ValSw4_1 (MH925091.1), which were all isolated using *V. harveyi* as the host, in both the DNA polymerase I and TerL trees. These four phages are clearly related to another branch, where four vibriophages were isolated from different *Vibrio* species. All eight vibriophages in the phylogenetic analysis were siphoviruses, with similar genome sizes (79.6–82.2 kb) and G + C contents (45.0–47.6%).

### Virion stability of R18L

**Figure 5:**
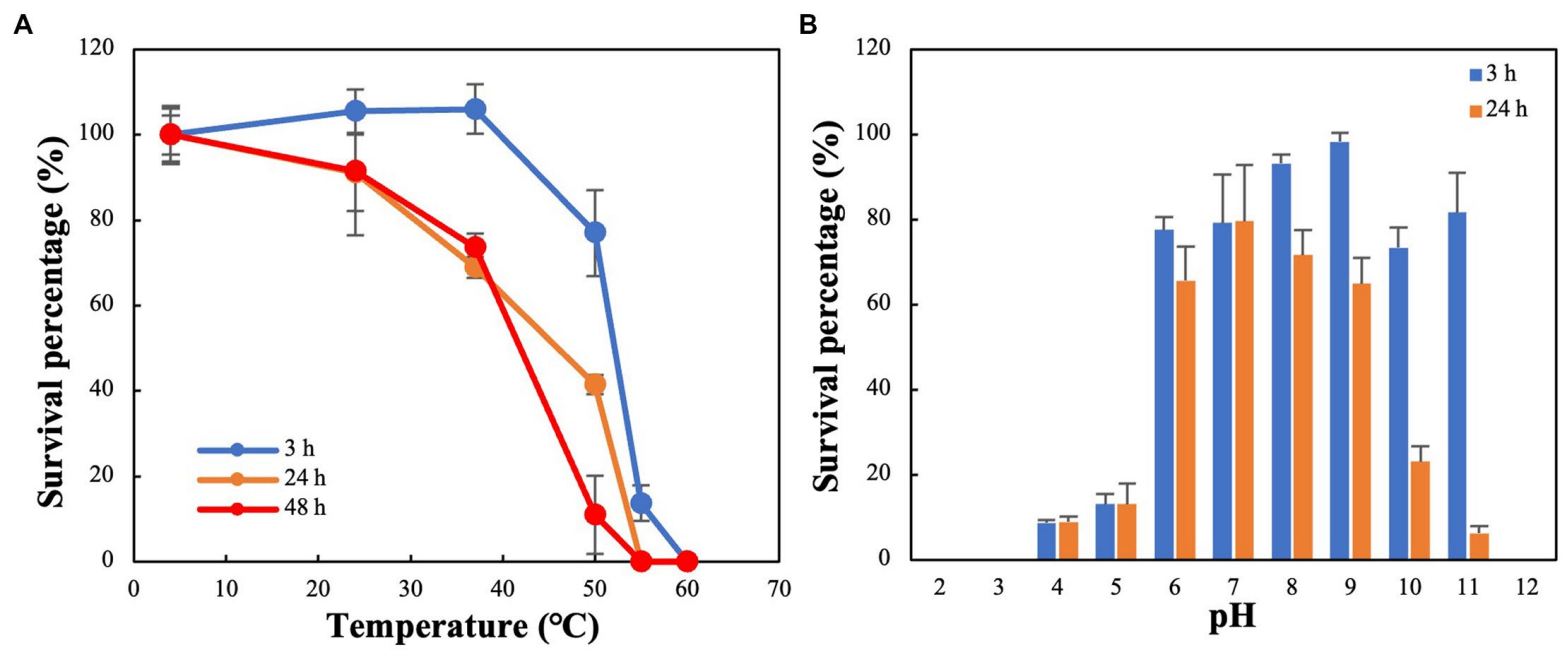
Stability of vibriophage vB_VhaS-R18L under different temperature **(A)** and pH **(B)** conditions. Data points represent the mean values and standard deviations of three independent experiments.

The thermal stability test showed that R18L was highly stable, with its activity remaining fairly constant at 4–40°C for 3 h (Figure 5A). Furthermore, greater than 50% of R18L phage remained active after incubation at 4–40°C for 24–48 h. The stability of R18L below 40°C simplifies the storage and transport requirements for this phage. Most phages become inactive when the temperature reaches 55°C, with survival percentages lower than 15% within 3 h. R18L did not show any activity when the temperature increased to 60°C, showing better thermal tolerance than previously reported vibriophages IME271 (40°C) (Li et al., 2019) and V-YDF132 (Kang et al., 2022). R18L exhibited stability over a wide range of pH (pH 6–11) for 3 h (Figure 5B). The survival percentage of phages decreased dramatically when the pH decreased to 5. When the incubation time reached 24 h, more than 60% of phages were still infective within the pH range 6–9, which indicated that R18L was relatively stable under such pH conditions.

## Conclusion

*Vibrio* is a major pathogen of various aquatic animals and causes vibriosis outbreaks in the aquaculture industry. Phage therapy is gaining increasing attention as a potentially effective strategy for controlling pathogenic bacteria. In this study, lytic phage R18L was isolated and characterized in terms of genomic organization, and phylogenetic and microbiological characteristics. R18L was able to infect bacteria across at least five pathogenic *Vibrio* species, thereby indicating its potential application as a biocontrol agent to control vibriosis. No antibiotic resistance, lysogeny-related, or virulence genes were detected in the R18L genome, suggesting the safety of this phage in biocontrol applications. Furthermore, R18L may be a good candidate for phage therapy because of its stability across a wide range of pH (6.0–11.0) and thermal (up to 55°C) conditions. In the field, one key feature of various *Vibrio* strains is their ability to form biofilm. Biofilm destruction by phages has been revealed to be more effective than antibiotics (Khalifa et al., 2016). Numerous experiments have been performed using single phages or phage cocktails against biofilms (Pires et al., 2017). However, the demonstration of the biofilm removal ability of R18L is necessary to determine its application in the biocontrol of vibriosis. Furthermore, other crucial questions that remain to be answered before field applications include testing the effectiveness of R18L in saline environments in future studies.

## Data availability statement

The datasets presented in this study can be found in online repositories. The names of the repository/repositories and accession number(s) can be found in the article/supplementary material.

## Author contributions

RZ, LC, and CA designed the project. ZL performed seawater sampling and phage isolation. LC, YT, ZL, and YY performed experiments and data analyses. LC, YT, and RZ wrote the manuscript. All authors contributed to the article and approved the submitted version.

## Funding

This study was supported by the National Key Research and Development Program of China (2021YFE0193000 and 2020YFA0608300) and National Natural Science Foundation of China (42188102 and 91951209).

## Conflict of interest

The authors declare that the research was conducted in the absence of any commercial or financial relationships that could be construed as a potential conflict of interest.

## Publisher’s note

All claims expressed in this article are solely those of the authors and do not necessarily represent those of their affiliated organizations, or those of the publisher, the editors and the reviewers. Any product that may be evaluated in this article, or claim that may be made by its manufacturer, is not guaranteed or endorsed by the publisher.

## Footnotes

^1^ https://blast.ncbi.nlm.nih.gov/Blast.cgi
